# A kinematic analysis of the hammer throw technique in elite female athletes: a comparative study between Chinese and international competitors

**DOI:** 10.3389/fphys.2025.1590350

**Published:** 2025-09-19

**Authors:** Weijie Liu, Yiling Ding, Ruihua He, Jianchen Li

**Affiliations:** ^1^ Capital University of Physical Education and Sports, Beijing, China; ^2^ China West Normal University, Nanchong, Sichuan, China; ^3^ Hebei University of Engineering, Handan, China

**Keywords:** kinematic, hammer throw technique, female athletes, throwing technique, Chinese and international competitors

## Abstract

**Purpose:**

This study aimed to examine the kinematic features of the hammer throw technique among elite female athletes worldwide, which is essential for advancing the development of women’s hammer throw in China. By examining the technical differences between Chinese female hammer throwers and their international counterparts, this research offers data support to help Chinese athletes further refine and optimise their throwing techniques, thereby strengthening the scientific basis of training in this discipline.

**Results:**

The study found that the average release velocity of Chinese athletes was lower than that of world-class athletes, with Zheng Wang recording the highest release velocity among the Chinese throwers. There was minimal difference in the average release angle between Chinese and international athletes. Apart from Zheng Wang, most Chinese athletes showed irregular timing in their average rotational phases, which negatively impacted the release phase. Notably, the average preliminary swing speed of Chinese athletes was markedly higher than that of international athletes, indicating a technical tendency towards active acceleration during the initial rotation phase. Additionally, the average trajectory length per turn among top Chinese athletes was generally longer than that of elite international athletes.

**Conclusion:**

The international elite female hammer throwers exhibit superior release-related indices and more effective double-support mechanics compared with their Chinese counterparts. These results highlight the central role of double-support coordination, trunk–pelvis coupling, and phase-specific timing in optimizing release velocity and overall performance. Targeted technical interventions focusing on these elements may help close the performance gap and advance elite-level outcomes.

## Introduction

Since women’s hammer throw was included in the World Athletics Championships in 1999, the sport has developed rapidly worldwide. In recent years, athletes like Poland’s Anita Włodarczyk and the United States’ DeAnna Price have delivered exceptional performances. Among them, Włodarczyk has retained a dominant position in women’s hammer throw, winning the gold medal at the 2016 Rio Olympic Games and setting a world record of 82.98 m, making her the only female hammer thrower in history to exceed the 80-m mark. Although China started developing the hammer throw event relatively late, it has advanced swiftly, producing exceptional athletes such as Yuan Gu, Wenxiu Zhang, Zheng Wang, Na Luo, and Jie Zhao (Dong, 2023). At the 2014 National Track and Field Throwing Championships in Chengdu, Chinese athlete Zheng Wang set a new Asian record with a throw of 77.68 m, a record that remains unbroken to this day. Additionally, she won a silver medal for the Chinese delegation at the 2021 Tokyo Olympic Games.

With the global advancement of women’s hammer throw, scientific research on its techniques has increasingly expanded. In the 1980s, (Dapena and Teves, 1982), carried out kinematic research on the hammer throw. In his 1982 study on the effects of air resistance after release, he showed that increased air resistance results in a decrease in throwing distance. His research from 1986 further revealed that increasing the proportion of double-support time during the acceleration phase could effectively enhance rotational speed and extend the hammer’s acceleration path (Panoutsakopoulos, 2006; Panoutsakopoulos, 2006) confirmed, through an analysis of the throwing techniques of 29 hammer throwers who participated in the World Athletics Championships and three Spanish National Championships, that hammer velocity was affected by the duration of the single- and double-support phases during the rotational process. When the hammer attained a certain velocity, the azimuth angle during the double-support phase was often smaller than at lower average velocities. Consequently, prolonging the double-support phase helped achieve a higher average velocity.

Regarding the key factors affecting hammer throw performance, both Chinese and international researchers have conducted biomechanical studies from various perspectives. The study (Panoutsakopoulos, 2006) concentrated on the final exertion phase, proposing that the excessive centrifugal force produced by the hammer’s high-speed flight results in considerable energy consumption. Therefore, athletes must keep a straight back, lock the left shoulder, and actively extend both legs to minimise energy loss. Bandou et al. (2006) discovered that the magnitude of acceleration during each turn was not strongly associated with throwing distance. However, athletes who could accelerate at higher rotational speeds and possessed the necessary physical strength and technical control were more likely to improve their performance.

In terms of specialised training, Hirose et al. (2017) conducted a study using an 8-kg hammer. After 4 weeks of intensive hammer training, the experimental group of 16 male athletes showed significant improvements in both throwing distance and release velocity, due to improvements in rotational speed. Furthermore, both the maximum centrifugal force during the double-support phase and the range of motion in the hip and knee joints of the lower limbs during rotation were improved, thereby confirming that heavy hammer training is an effective method for enhancing throwing distance.

Regarding the technical characteristics of the women’s hammer throw, studies have demonstrated notable differences compared to the men’s event. Unlike men, who use heavier equipment, women’s hammer throw focuses on rotational speed and release velocity techniques (Shi and Dong, 2022). Murofushi et al. (2007) further observed that top female hammer throwers spent less time in the rotational phase and had shorter overall cycle durations. They effectively allocated the proportions of single- and double-support phases, maintained a steady rotational rhythm, and achieved smooth acceleration. During the double-support phase, they maintained balance by lowering their centre of gravity and improved hammer control, thereby establishing a cohesive athlete-hammer system that created favourable conditions for the final release. The present evolution of hammer throw techniques emphasises a trend towards shorter rotation periods and higher release speeds.

The technological progress in women’s hammer throw plays a strategic role in boosting the overall competitiveness of athletics in China and globally. Given the current global trend towards ‘short-cycle rotation and high release velocity’ in women’s hammer throw, as well as the objective differences between Chinese and international athletes in key phases such as support phase control and athlete-hammer coordination. The aim of this study was to systematically analyse the differential characteristics of core technical elements—such as rotational rhythm, force sequencing, and centre of gravity control—between elite Chinese throwers and world-class athletes through a precise comparison of kinematic parameters. The results are expected to deliver personalised technical optimisation plans for Chinese female hammer throwers and provide scientific backing for training practices focused on enhancing acceleration efficiency during the double-support phase and improving technical stability at high-speed rotation, thereby advancing the refinement and professionalisation of the training system in this discipline.

## Materials and methods

### Tested subjects

This study examined the throwing techniques of top female hammer throwers from both China and abroad as the main research subjects. This study selected a total of 14 athletes and 16 throwing techniques as the research sample. The sample was chosen based on the top six performances by athletes in the women’s hammer throw at the National Athletics Championships and Olympic Trials held on 11 June 2021. Additionally, the top eight athletes’ best results from the women’s hammer throw event at the IAAF World Challenge Nanjing meeting on 21 May 2019, were collected. The fundamental details about the athletes are provided in Tables 1, 2. Specifically, Zheng Wang 1 and Na Luo 1 correspond to data from the 2021 National Athletics Championships and Olympic Trials, while Zheng Wang 2 and Na Luo 2 relate to data from the 2019 IAAF World Challenge.

**TABLE 1:** Personal information of elite Chinese athletes.

Athlete	Age	Height (m)	Weight (kg)	Competition PB (m)	Personal best (m)
Zheng Wang 1	33	1.74	103	73.38	77.68
Zheng Wang 2	31	1.74	105	75.27	77.68
Na Luo 1	28	1.75	88	68.78	75.02
Na Luo 2	26	1.75	88	71.6	75.02
Weilu Huang	22	1.79	99	69.96	70.16
Fan Zhao	23	1.76	72	66.82	66.45
Tingting Liu	30	1.78	93	66.81	73.80
Jiangyan Li	22	1.80	90	65.81	74.47

Note: Data sourced from the official World Athletics website.

**TABLE 2:** Personal information of elite international athletes.

Athlete	Age	Height (m)	Weight (kg)	Competition PB (m)	Personal best (m)
DeAnna PRICE (US)	26	1.73	109	74.21	78.24
Anita WLODARCZYK (Poland)	34	1.78	95	73.64	82.98
Brooke ANDERSEN (US)	24	1.78	88	73.4	80.17
Hanna SKYDAN (Azerbaijan)	27	1.83	105	72.92	77.10
Gween BERRY (US)	30	1.73	88	72.79	77.78
Joanna FIODOROW (Poland)	30	1.70	90	72.34	76.35

Note: Data sourced from the official World Athletics website.

### Mesuremant methods

#### Three-dimensional close-range fixed camera method

**FIGURE 1:**
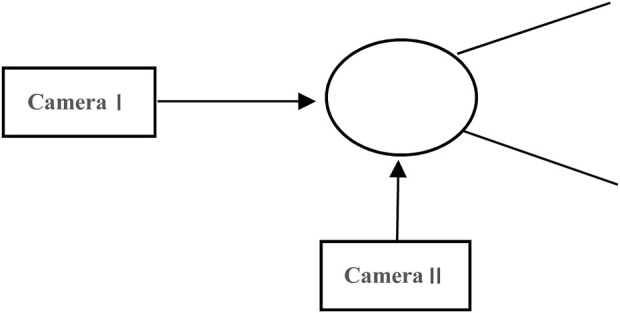
On-site camera setup diagram. CameraⅠ. CameraⅡ.

This study carried out fixed-point filming of the women’s hammer throw finals at the 2019 IAAF World Challenge Nanjing meeting and the 2021 National Athletics Championships and Olympic Trials. Two JVC high-speed cameras were used for filming, with the Peak3D digital calibration frame from the United States acting as the reference framework. Before the competitions, the two JVC high-speed cameras were placed about 20 m behind the throwing circle and 6 m along the extension of the circle’s centreline. Both cameras were positioned at a height of 1.3 m, with their main optical axes oriented at a right angle to each other. The filming frequency was set at 100 Hz. The Peak3D digital calibration frame was recorded once before the official competition and once after it concluded, with its geometric centre positioned 1.3 m directly above the centre of the hammer throwing circle. The specific arrangement of the recording site is shown in Figure 1.

#### Three-dimensional kinematic analysis method

This study used the SignalTEC Video Rapid Feedback Analysis System—a sophisticated three-dimensional biomechanical analysis tool—to examine the hammer throw techniques of 14 elite athletes from both China and abroad. The focus was on the top six athletes’ best performances in the women’s hammer throw final at the 2021 National Athletics Championships and Olympic Trials, as well as the top eight athletes from the 2019 IAAF World Challenge Nanjing meeting. Additionally, the study included the centre of mass trajectories from 14 throws by 12 notable female hammer throwers, using the 21-point human body model proposed by Hideharu Matsui from Japan. Using the athletes’ joint points and additional marker points, the collected images were manually digitised and processed for data analysis. Finally, the raw data obtained from the analysis was smoothed using a low-pass digital filtering method.

### Data analysis

This study used Excel 2016 and SPSS 23.0 statistical software to organise and compare the kinematic parameters of the athletes’ throwing techniques to gather the necessary data. Pearson correlation analysis was carried out to investigate the relationship between the kinematic parameters of the hammer throw and performance outcomes. The level of significance was set at P < 0.05, indicating a statistically significant correlation.

### Definition of relevant indicators

This study defined several relevant indicators: T1 refers to the period from when the hammer reaches its lowest point at the end of the preliminary swings to the first lift-off of the right foot and its subsequent first ground contact. T2, T3, and T4 represent the durations from the right foot’s lift-off to ground contact during the second, third, and fourth rotations, respectively. D signifies the period from the right foot’s fourth ground contact until the hammer is released. R↑ and R↓ signify the moments when the right foot lifts off and makes contact with the ground, respectively. The release parameters consist of the velocity, angle, and height of the hammer at the moment of release. Time parameters include the total rotation time, the duration of each turn, and the length of the final exertion phase. Velocity parameters include the hammer’s speed during the initial swings, each rotation, and the final exertion phase. Trajectory parameters include the total length of the hammer’s path during rotation as well as the length of each turn. The shoulder-hammer angle is defined as the angle between the line connecting the centres of both shoulder joints and the horizontal projection of the left side of the chain.

## Results

### Analysis of release parameter characteristics of Chinese and international hammer throwers

**FIGURE 2:**
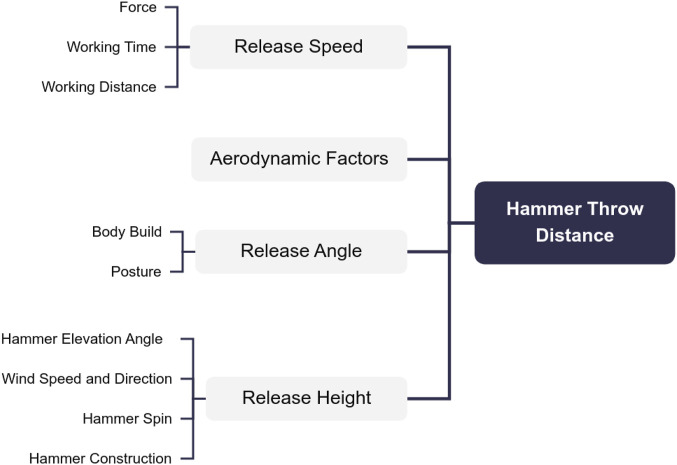
Factors influencing hammer throw distance.

As shown in Figure 2, the factors affecting the distance of a shot put throw include release speed, release height, and release angle.

#### Release velocity analysis

According to the equations of projectile motion, when all other conditions remain unchanged, the throwing distance is directly proportional to the square of the initial release velocity. Therefore, the initial release velocity is the most crucial factor determining the flight distance of the hammer (Zhang, 2017). Among the various factors affecting hammer throw distance—including release height, release angle, and release velocity—release velocity is the most critical. It indicates the athlete’s kinetic energy and is the only parameter that needs to be maximised for optimal performance. Table 3 shows a strong positive correlation between release velocity and throwing distance (r = 0.782, P = 0.001). As shown in Table 4, the average release velocity of elite Chinese athletes was 27.09 ± 1.44 m/s, which is lower than the 27.51 ± 1.04 m/s recorded by world-class athletes. Notably, Zheng Wang achieved a release velocity of 29.31 m/s, the highest among all athletes, and a high release velocity is a notable feature of her technique. However, when comparing performances from two competitions, Zheng Wang’s release velocity during the 2021 National Athletics Championships and Olympic Trials (Zheng Wang 1) was considerably lower than that in the 2019 IAAF World Challenge (Zheng Wang 2), suggesting potential for improvement in her final exertion phase. The release velocity of Chinese athlete Jiangyan Li was lower than that of her domestic and international peers, mainly due to her velocity and trajectory parameters during the final exertion phase. The study found that Jiangyan Li’s duration of the final exertion phase was 2.80 s, notably longer than Zheng Wang’s 2.20 s, indicating lower movement efficiency. Furthermore, analysis of the velocity parameters during the final exertion phase showed that Jiangyan Li’s peak implement velocity was 24.45 m/s, which is relatively low and unfavourable for increasing the initial release velocity of the hammer. Therefore, enhancing the initial release velocity should become a main focus in future training of Chinese hammer throwers.

**TABLE 3:** Correlation between release parameters and throwing performance for Chinese and foreign athletes (N = 14).

Release parameter	Mean ± standard deviation	Correlation coefficient (r)	P-value
Release Speed	27.27 ± 1.26	0.782**	0.001
Release Angle	37.65 ± 2.33	0.241	0.406
Release Height	1.50 ± 0.19	−0.582*	0.029

Statistical significance: * - p < 0.05, **- p < 0.01, *** - p < 0.001.

**TABLE 4:** Release parameters of Chinese and foreign athletes.

Athlete	Release speed (m/s)	Release angle (°)	Release height (m)
Zheng Wang 1	28.76	39.67	1.61
Zheng Wang 2	29.31	39.04	1.41
Na Luo 1	27.53	36.15	1.59
Na Luo 2	26.65	36.87	1.31
Weilu Huang	27.43	37.11	1.78
Fan Zhao	26.12	32.42	1.43
Tingting Liu	25.87	42.63	1.90
Jiangyan Li	25.11	36.99	1.69
X±SD	27.09 ± 1.44	37.61 ± 2.97	1.59 ± 0.19
DeAnna PRICE	29.01	37.83	1.46
AnitaWLODARCZYK	28.46	39.58	1.51
Brooke ANDERSEN	27.44	38.54	1.33
Hanna SKYDAN	27.09	37.36	1.38
Gween BERRY	26.22	37.21	1.36
Joanna FIODOROW	26.86	35.70	1.25
X±SD	27.51 ± 1.04	37.70 ± 1.31	1.38 ± 0.09

### Release angle analysis

The release angle had a notable effect on the hammer’s flight time. As shown in Table 3, the average release angle for Chinese athletes was 37.6° ± 3°, while that for international athletes was 37.7° ± 1°, indicating a slight difference between the two groups. However, it is notable that Chinese athletes showed significant variation in their release angles. Tingting Liu’s release angle was notably higher than that of her peers, reaching 42.6°. This was mainly caused by a ‘back-hammer’ movement during her final two rotations, combined with insufficient compression and overextension of her left knee joint. In contrast, Fan Zhao’s release angle was considerably lower, at only 32.4°, primarily because her left foot rotation was inadequate during the rotational phase. During each turn, as her right foot left the ground, her left toe failed to align properly with the throwing direction. Additionally, a ‘hammer pulling the body’ phenomenon was observed during the final two rotations, indicating that the ‘athlete-hammer unity’ technique was not optimally achieved. This caused a reduced release angle, which negatively impacted the throwing distance.

### Release height analysis

The release height indicates the athlete’s capacity to consistently exert force on the hammer. Usually, a higher release point enhances the athlete’s performance. The optimal release height happens when the athlete is at the edge of the throwing circle and is affected by both their physical traits and technical skill. As the release height increases, the throwing distance also increases, providing taller athletes with a natural advantage over their shorter counterparts. A comparison of the physical features of Chinese and international athletes showed that Zheng Wang from China, DeAnna Price from the United States, and Anita Włodarczyk from Poland all effectively used their body characteristics to optimise release height. Notably, although Zheng Wang did not have the most advantageous physical attributes or height advantage, she maximised her release height and extended the duration of her final exertion phase, positively influencing her throwing distance. In contrast, Fan Zhao and Tingting Liu, despite possessing advantageous physical conditions, did not fully utilise their technical potential. Fan Zhao’s release height was the lowest among the six athletes, at just 1.43 m. This lower release height caused a smaller release angle, which impaired the acceleration of the implement during the final exertion phase. Therefore, Tingting Liu and Fan Zhao need to further enhance their technical execution of release height during this phase to optimise their throwing performance.

### Analysis of time parameter characteristics in hammer throw techniques between Chinese and international athletes

Modern hammer throw techniques feature high speed and a fast rhythm. The swift pace of movements and shorter rotation periods are crucial for improving athletic performance. Athletes are expected to actively increase their speed during the rotational process while gradually decreasing the time taken for each turn (Liu et al., 2008). Both Chinese and international athletes have adopted the four-turn rotational technique. As shown in Table 5. With the same rotational radius, a significant negative correlation exists between total rotation time and throwing performance (r = −0.751, P = 0.002). This indicates that shorter rotation times correspond to higher rotational speeds, which in turn increase the hammer’s velocity and extend the throwing distance. As shown in Table 6 the total rotation times for Chinese athletes were 2.16 s, 2.07 s, 2.34 s, 2.25 s, 2.28 s, 2.30 s, 2.50 s, and 2.36 s, while the total rotation times for international athletes were 2.13 s, 2.19 s, 2.24 s, 2.35 s, 2.30 s, and 2.33 s. The average total rotation time for international athletes (2.25 ± 0.09 s) was shorter than that for Chinese athletes (2.28 ± 0.13 s). Among all athletes, Zheng Wang achieved the shortest total rotation time at 2.07 s, making her the fastest female hammer thrower in terms of rotational speed. In contrast, Tingting Liu had the longest total rotation time, which resulted in generally slower rotational speeds that negatively impacted her release performance. The women’s hammer throw is classified as a speed-strength event, where the main factor affecting velocity is the acceleration rhythm. An effective acceleration rhythm is usually marked by a steady decrease in rotation time and a consistent increase in implement velocity (Zhi et al., 2007). Comparative analysis showed that the average rotation time of both Chinese and international athletes gradually decreased with each turn, aligning with the modern women’s hammer throw technique principle of ‘early hammer reception and early release’. However, apart from Zheng Wang, Chinese athletes showed irregular rhythm patterns, especially Fan Zhao, Tingting Liu, and Jiangyan Li, who exhibited prolonged rotation times during the second turn. This was probably due to not relaxing the arm enough during the transition from the preliminary swings to the rotational phase, which led to weaker control of the implement, a smaller hammer rotation radius, disrupted rotational rhythm, and hindered acceleration. Among these athletes, Tingting Liu had the longest total rotation time, resulting in a generally slower rotational speed that negatively affected the final release. Additionally, Jiangyan Li’s rotation time suddenly decreased during the third turn, indicating a hurried effort to speed up; however, this disrupted rhythm ultimately hindered the perfect hammer release.

**TABLE 5:** Correlation between rotation time, final force time, total time, and performance for Chinese and foreign athletes.

Rotation turn	Mean ± standard deviation (s)	Correlation coefficient (r)	P-value
T1	0.53 ± 0.02	0.068	0.818
T2	0.55 ± 0.05	−0.812***	0.000
T3	0.49 ± 0.03	0.020	0.946
T4	0.46 ± 0.31	−0.275	0.341
D	0.24 ± 0.04	−0.890***	0.000
Total Time	2.27 ± 0.11	−0.751***	0.002

Statistical significance: * - p < 0.05, **- p < 0.01, *** - p < 0.001.

**TABLE 6:** Rotation time per turn, final force time, and total time parameters for Chinese athletes.

Athlete	T1 (s)	T2 (s)	T3 (s)	T4 (s)	D (s)	Total time (s)
Zheng Wang 1	0.52	0.5	0.5	0.42	0.22	2.16
Zheng Wang 2	0.54	0.49	0.44	0.41	0.19	2.07
Na Luo 1	0.54	0.58	0.5	0.42	0.3	2.34
Na Luo 2	0.55	0.52	0.48	0.46	0.24	2.25
Weilu Huang	0.52	0.54	0.48	0.48	0.26	2.28
Fan Zhao	0.5	0.58	0.46	0.44	0.32	2.3
Tingting Liu	0.56	0.64	0.52	0.5	0.28	2.5
Jiangyan Li	0.52	0.6	0.46	0.5	0.28	2.36
X±SD	0.53 ± 0.02	0.56 ± 0.05	0.48 ± 0.03	0.45 ± 0.04	0.26 ± 0.04	2.28 ± 0.13
DeAnna PRICE	0.52	0.49	0.46	0.45	0.21	2.13
Anita WLODARCZYK	0.51	0.5	0.49	0.48	0.21	2.19
Brooke ANDERSEN	0.56	0.54	0.48	0.48	0.18	2.24
Hanna SKYDAN	0.54	0.55	0.53	0.49	0.24	2.35
Gween BERRY	0.5	0.57	0.54	0.49	0.2	2.3
Joanna FIODOROW	0.56	0.58	0.51	0.48	0.2	2.33
X±SD	0.53 ± 0.03	0.54 ± 0.04	0.50 ± 0.03	0.47 ± 0.01	0.21 ± 0.02	2.25 ± 0.09
Max	0.56	0.64	0.54	0.5	0.32	2.5
Min	0.5	0.49	0.44	0.41	0.18	2.07

### Analysis of velocity parameters at each rotational phase for Chinese and international athletes

The initial release velocity of the hammer mainly depended on the incremental increase in rotational speed during each turn and the improvement of the peak rotational speed achieved per turn. This increase followed a rhythmically progressive pattern with each successive turn (Han, 2010). As shown in Table 7, the hammer velocity during the preliminary swings was significantly negatively correlated with throwing performance (r = −0.551, P = 0.041). In contrast, the hammer velocities during the third turn, fourth turn, and final exertion phase were significantly positively correlated with performance (P < 0.05). The initial swings contributed the most to the increase in velocity, accounting for approximately 50% of the final release velocity. An appropriate initial swing velocity helped establish a more compact rotational rhythm, enabling the smooth execution of the movement. Therefore, managing the initial swings was essential for a smooth transition into the first turn of rotation. According to Table 8, the average preliminary swing velocity of Chinese athletes (16.20 ± 1.52 m/s) was notably higher than that of international athletes (13.57 ± 0.96 m/s), reflecting a technical tendency among Chinese athletes to actively accelerate when entering the first turn. Among all athletes, American athlete DeAnna Price recorded the lowest preliminary swing velocity at 12.22 m/s, making her the slowest among all competitors. A lower preliminary swing velocity disrupted the subsequent rotational trajectory and rhythm, emphasising the need for targeted training to strengthen the initial swing phase and improve speed-strength capabilities. During the rotational phase, the hammer velocity of both Chinese and international athletes generally showed a fluctuating upward trend, consistent with the technical characteristics of modern women’s hammer throw. However, Chinese athlete Weilu Huang showed a negative velocity increase during the final exertion phase, leading to a lower peak velocity, which ultimately impacted the release velocity.

**TABLE 7:** Correlation between hammer speed in each rotation phase and throwing performance for Chinese and foreign athletes.

Rotation turn	Mean ± standard deviation (m/s)	Correlation coefficient (r)	Rotation turn
the preliminary swings	15.07 ± 1.85	−0.551*	0.041
T1	17.38 ± 1.67	−0.056	0.848
T2	21.44 ± 1.26	0.018	0.952
T3	24,48 ± 1.42	0.542*	0.045
T4	26.41 ± 1.41	0.833***	0.000
D	27.03 ± 1.41	0.820***	0.000

Statistical significance: * - p < 0.05, **- p < 0.01, *** - p < 0.001.

**TABLE 8:** Hammer speed parameters in each rotation phase for Chinese and foreign athletes.

Athlete	Pre-swing (m/s)	T1 (m/s)	T2 (m/s)	T3 (m/s)	T4 (m/s)	D (m/s)
Zheng Wang 1	17.16	20.62	23.04	27.25	28.26	28.31
Zheng Wang 2	14.77	17.9	23.55	25.71	29.12	29.31
Na Luo 1	16.4	17.12	21.76	24.99	26.9	27.52
Na Luo 2	14.29	16.52	22.61	25.2	26.15	26.65
Weilu Huang	18.82	20.56	23.77	24.73	25.92	25.62
Fan Zhao	17.18	17.46	21.96	23.38	24.34	25.52
Tingting Liu	14.95	15.4	19.16	23.01	25.15	26.03
Jiangyan Li	16.02	18.58	21.88	22.88	23.9	24.45
X±SD	16.20 ± 1.52	18.02 ± 1.84	22.22 ± 1.45	24.64 ± 1.50	26.22 ± 1.82	26.68 ± 1.61
DeAnna PRICE	12.22	16.46	19.92	25.59	26.99	29.01
Anita WLODARCZYK	14.98	16.87	20.28	26.31	27.38	28.46
Brooke ANDERSEN	13.63	16.42	21.22	23.91	27.3	27.44
Hanna SKYDAN	13.07	14.88	18.72	22.76	26.1	27.09
Gween BERRY	13.26	16.49	21.74	24	25.82	26.22
Joanna FIODOROW	14.26	17.97	20.53	22.98	26.38	26.86
X±SD	13.57 ± 0.96	16.52 ± 0.99	20.40 ± 1.05	24.26 ± 1.42	26.66 ± 0.65	27.51 ± 1.04

### Analysis of trajectory length parameters at each rotational phase for Chinese and international athletes

The initial release velocity of the hammer is a vital factor affecting throwing performance, mainly influenced by the angular velocity of the hammer’s rotation and the radius of rotation (Brice et al., 2011). The length of the rotation radius mainly depends on the trajectory length of the hammer during each technical phase; therefore, the hammer’s trajectory length is an important indicator for measuring hammer velocity.

Table 9 shows that the total trajectory length during the rotation process is strongly negatively correlated with throwing performance (r = −0.814, P = 0.000). As shown in Table 10, the average trajectory length per turn for elite Chinese athletes (46.55 ± 3.85 m) is markedly higher than that of foreign athletes (41.19 ± 0.66 m). During rotation, a larger hammer’s radius results in a longer trajectory per turn, indicating an extended working distance and increased energy transfer to the hammer at the moment of release, which significantly contributes to the hammer’s release velocity. Comparative data analysis shows that in the two most recent competitions, Zheng Wang’s trajectory length from the end of the preliminary swings to the first turn was slightly shorter than during her 2014 Asian record in Chengdu, China, where her trajectory length in the first turn was 9.63 m (Dong, 2017). The decrease in trajectory length during the initial turn in the two latest competitions was attributed to Zheng Wang’s technical movements during the transition from the preliminary swings to the first turn. As a member of the scientific and technical support team for hammer throw research, multiple analyses were carried out on Zheng Wang’s training and competition performances. It was observed that Zheng Wang, along with several other Chinese female hammer throwers, showed a tendency for early lower limb acceleration during the transition from preliminary swings to the rotational phase, which was accompanied by shoulder tension and insufficient relaxation of the upper limbs. This technical issue caused a shortened trajectory length during the first turn, disrupting the overall rotational rhythm of the hammer and ultimately impacting the release velocity. Compared to foreign athletes, some Chinese athletes showed differences in trajectory length across various turns during the rotational phase. Although Chinese athlete Weilu Huang achieved an overall ideal total trajectory length, the average radius in the first turn was relatively small, resulting in a “small-large-small-large” pattern throughout the entire rotational sequence. Previous analyses of velocity parameters showed that Weilu Huang’s initial swing velocity was quite high, which caused unstable velocity control when entering the first turn and disturbed the overall rhythm. In this competition, Tingting Liu’s average radii for each turn were 1.31 m, 1.87 m, 1.82 m, and 1.86 m, displaying a “small-large-large-large” pattern. Although her rotational stability improved, the radius in the first turn still remained relatively short. Unlike Weilu Huang, Tingting Liu’s technical issue arose from her slower initial swing velocity, which led to a reduced hammer velocity during the first turn and consequently impacted the trajectory length at this stage. The relationship between velocity and trajectory shows that the rotation radius and movement amplitude in each phase directly affect the hammer’s linear velocity during rotation. A technical analysis of these two athletes concluded that both excessively fast and excessively slow preliminary swing velocities negatively impact the rotation radius and trajectory length in the first turn. Therefore, Weilu Huang and Tingting Liu should focus on improving their transition techniques from the preliminary swings to the first turn in future training to attain better velocity increments.

**TABLE 9:** Correlation between trajectory length per rotation and throwing performance for Chinese and foreign athletes.

Rotation turn	Mean ± standard deviation (m)	Correlation coefficient (r)	P-value
T1	8.85 ± 0.75	−0.120	0.684
T2	11.42 ± 0.91	−0.809***	0.000
T3	11.12 ± 0.79	−0.379	0.182
T4	11.27 ± 0.62	−0.503	0.067
Total	44.25 ± 3.97	−0.814***	0.000

Statistical significance: * - p < 0.05, **- p < 0.01, *** - p < 0.001.

**TABLE 10:** Trajectory length parameters per rotation for Chinese and foreign athletes.

Athlete		T1 (m)	T2 (m)	T3 (m)	T4 (m)	Total (m)
Zheng Wang 1	Trajectory Length	9.41	11.14	12.56	11.39	46.42
	Average Radius	1.44	1.70	1.92	1.73	
Zheng Wang 2	Trajectory Length	8.9	10.88	10.22	10.96	40.96
	Average Radius	1.42	1.73	1.63	1.75	
Na Luo 1	Trajectory Length	8.57	11.78	11.98	10.84	47.66
	Average Radius	1.31	1.82	1.84	1.66	
Na Luo 2	Trajectory Length	8.37	10.68	10.52	10.79	40.36
	Average Radius	1.33	1.70	1.68	1.72	
Weilu Huang	Trajectory Length	9.93	12.84	12.07	12.35	50.53
	Average Radius	1.55	1.97	1.85	1.90	
Fan Zhao	Trajectory Length	8.79	12.41	11.12	11.12	48.03
	Average Radius	1.35	1.91	1.70	1.70	
Tingting Liu	Trajectory Length	8.53	12.12	11.86	12.14	48.55
	Average Radius	1.31	1.87	1.82	1.86	
Jiangyan Li	Trajectory Length	9.50	12.98	11.04	12.25	49.88
	Average Radius	1.46	2.00	1.69	1.88	
X±SD		9.00 ± 0.55	11.85 ± 0.88	11.42 ± 0.82	11.48 ± 0.66	46.55 ± 3.85
DeAnna PRICE	Trajectory Length	9.58	10.89	10.11	11.22	41.8
	Average Radius	1.53	1.73	1.61	1.79	
Anita WLODARCZYK	Trajectory Length	7.78	10.72	11.26	11.42	41.18
	Average Radius	1.24	1.71	1.79	1.82	
Brooke ANDERSEN	Trajectory Length	8.98	9.73	10.33	11.07	40.11
	Average Radius	1.43	1.55	1.64	1.76	
Hanna SKYDAN	Trajectory Length	7.73	11.18	11.26	11.32	41.49
	Average Radius	1.23	1.78	1.79	1.80	
Gween BERRY	Trajectory Length	7.89	11.35	11.24	10.26	40.74
	Average Radius	1.26	1.81	1.79	1.63	
Joanna FIODOROW	Trajectory Length	9.94	11.18	10.09	10.58	41.79
	Average Radius	1.58	1.78	1.61	1.68	
X±SD		8.65 ± 0.98	10.84 ± 0.59	10.72 ± 0.59	10.97 ± 0.46	41.19 ± 0.66

### Analysis of shoulder-hammer angle parameters at right foot ground contact (R↓) and take-off (R↑) moments during each turn in Chinese and international athletes

In the hammer throw, the angle formed between the line connecting the athlete’s shoulder joints and the horizontal projection of the hammer wire is called the shoulder-hammer angle. This measurement is commonly used to evaluate the relationship between the athlete and the implement (Liang, 2008). In modern women’s hammer throw techniques, the shoulder-hammer angle at the moment when the athlete’s right foot lifts off and enters the single-support phase tends to be just under 90° (Dong et al., 2014). This adjustment allowed female hammer throwers to better use their lower limb strength during the throw and effectively perform the ‘surpassing the implement’ movement (Liu, 2002). As a result, it lengthened the double-support phase while shortening the single-support phase (Wang and Wu, 2013). As shown in Table 11, apart from the moment of right foot take-off in the first turn, Chinese athletes displayed larger average shoulder-hammer angles than their international counterparts at the moments of right foot contact with the ground and take-off in the subsequent turns. An excessively large shoulder-hammer angle during the hammer throw is likely to cause a ‘back-hammer’ movement, which considerably decreases the hammer’s rotation radius during the rotational phase and negatively impacts its rotational velocity (Dong and Tian, 2011). Chinese athlete Zheng Wang kept shoulder-hammer angles below 90° at the moment of right foot take-off during all four turns. This technique helped the use of lower limb strength to perform the “surpassing the implement” movement, reducing the duration of the single-support phase. Additionally, Zheng Wang kept her shoulder-hammer angle close to 90° at right foot ground contact, ensuring stability and enabling effective hammer acceleration. In contrast, Tingting Liu and Jiangyan Li showed considerable fluctuations in shoulder-hammer angles during right foot take-off and ground contact in each turn, especially in the first and second turns, where their angles were relatively small (74.42° and 64.92°, respectively) as shown in Table 12. This caused incorrect “hammer-leading-body” rotations, hindering them from attaining the modern “athlete-hammer unity” technique. Going forward, they should focus on improving the stability and coordination of their initial swings and rotations. Chinese athlete Fan Zhao and American athlete Gwen Berry exhibited smaller fluctuations in shoulder-hammer angles during the four rotations, which impeded the maintenance of a stable rhythm. Video analysis indicated that this instability partly resulted from their somewhat lateral stance positions within the throwing circle. Excessively large shoulder-hammer angles caused incorrect “back-hammer” movements, impairing hammer acceleration during the double-support phase and disrupting overall rotational efficiency.

**TABLE 11:** Analysis of shoulder-wire angle parameters at R↑ and R↓ moments per rotation for Chinese and foreign athletes (°).

Athletes	First turn (°)		Second turn (°)		Third turn (°)		Fourth turn (°)	
	R↑	R↓	R↑	R↓	R↑	R↓	R↑	R↓
Zheng Wang 1	85.73	95.83	87.11	97.18	85.51	93.11	82.94	91.06
Zheng Wang 2	83.54	89.11	81.35	91.91	78.26	89.2	82.79	90.62
Na Luo 1	83.42	86.53	81.57	84.66	92.24	83.09	107.96	85.57
Na Luo 2	74.33	89.46	81.55	88.06	92.17	88.08	101.63	92.45
Weilu Huang	94.49	95.18	93.6	105.45	93.56	99.52	87.01	108.74
Fan Zhao	80.24	95.72	85.82	114.21	96.26	105.38	94.53	104.15
Tingting Liu	74.42	100.99	75.35	101.11	80.17	95.2	93.58	86.31
Jiangyan Li	64.92	101	78.73	108.23	89.37	108.79	90.91	112.26
X±SD	80.14 ± 8.93	94.23 ± 5.42	83.14 ± 5.61	98.85 ± 10.29	88.44 ± 6.52	95.29 ± 8.82	92.67 ± 8.83	96.39 ± 10.42
DeAnna PRICE	77.91	87.33	75.35	86.06	79.2	96.04	69.73	84.81
AnitaWLODARC	90.9	92.11	78.75	89.51	92.72	95.92	84.53	96.93
Brooke ANDERSEN	80.17	94.23	87.75	97.17	77.62	89.51	86.99	104.06
Hanna SKYDAN	82.14	94.9	86.9	98.82	86.38	102.08	82.22	95.92
Gween BERRY	87.45	96.53	82.51	99.62	88.08	97.21	79.42	99.81
Joanna FIODOROW	72.57	85.76	65.59	77.48	67.74	88.38	68.43	82.22
X±SD	81.86 ± 6.60	91.81 ± 4.35	79.48 ± 8.28	91.44 ± 8.74	81.86 ± 8.96	94.86 ± 5.11	78.53 ± 7.76	93.96 ± 8.61

**TABLE 12:** Correlation between shoulder-wire angle at R↑ and R↓ moments per rotation and throwing performance for Chinese and foreign athletes (N = 14).

Rotation turn	Mean ± standard deviation (°)	Correlation coefficient (r)	P-value
First Turn R↑	80.87 ± 7.77	0.418	0.137
First Turn R↓	93.19 ± 4.96	−0.526	0.053
Second Turn R↑	81.57 ± 6.85	−0.003	0.993
Second Turn R↓	95.68 ± 10.04	−0.555*	0.039
Third Turn R↑	85.66 ± 8.05	−0.407	0.148
Third Turn R↓	95.11 ± 7.21	−0.439	0.116
Fourth Turn R↑	86.62 ± 10.85	−0.583*	0.029
Fourth Turn R↓	95.35 ± 9.41	−0.341	0.233

Statistical significance: * - p < 0.05, **- p < 0.01, *** - p < 0.001.

## Discussion

The present study systematically analyzed the kinematic differences in hammer throw techniques between elite Chinese and international female athletes, with the aim of providing targeted technical optimization references for Chinese athletes. By integrating our findings with existing literature, we further clarify the technical characteristics and improvement directions of women’s hammer throw.

### Release velocity: core determinant of performance

Our results confirmed that release velocity is the most critical factor affecting throwing distance (r = 0.782, p = 0.001), which is consistent with the conclusion of Zhang (2017) that “release velocity has the highest correlation with performance among all release parameters”. Notably, the average release velocity of Chinese athletes (27.09 ± 1.44 m/s) was slightly lower than that of international athletes (27.51 ± 1.04 m/s), except for Zheng Wang, whose maximum release velocity (29.31 m/s) reached the international top level. This discrepancy may be attributed to the “time loss” in the final exertion phase of most Chinese athletes (e.g., Jiangyan Li’s 2.80 s vs Zheng Wang’s 2.20 s), which is consistent with Brice et al. (2011)’s view that “inefficient energy transfer in the final phase directly reduces release velocity”. Rojas Ruiz and Gutiérrez Dávila (2009) emphasized that prolonging the double-support phase can enhance velocity accumulation; our data supports this, as Chinese athletes with irregular rotation rhythms (e.g., Fan Zhao) had shorter double-support phases, leading to lower release velocities.

### Rotational Rhythm: stability determines acceleration efficiency

The total rotation time showed a significant negative correlation with performance (r = −0.751, p = 0.002), which aligns with Liu et al. (2008)’s assertion that “shorter rotation time with increasing speed per turn is a hallmark of excellent technique”. International athletes maintained a steady reduction in rotation time from T1 to T4, while most Chinese athletes (except Zheng Wang) exhibited irregular rhythms, particularly prolonged T2 (e.g., Tingting Liu’s 0.64s). This phenomenon is similar to the “rhythm disruption” observed by Murofushi et al. (2007) in sub-elite athletes, who attributed it to “imbalanced single- and double-support ratios”. Our further analysis found that the excessively high preliminary swing speed of Chinese athletes (16.20 ± 1.52 m/s) may be a key cause, as it disrupts the transition from swing to rotation—supporting Bandou et al. (2006)’s finding that “uncontrolled initial acceleration impairs rotational stability”.

### Trajectory length and preliminary swing: a balanced technical paradox

The total trajectory length was negatively correlated with performance (r = −0.814, p = 0.000), but Chinese athletes had longer average trajectories (46.55 ± 3.85 m) than international athletes (41.19 ± 0.66 m). This “longer but less efficient” phenomenon contradicts Panoutsakopoulos (2006)’s conclusion that “longer trajectory length contributes to velocity accumulation”, indicating that trajectory stability is more critical than length. Our data showed that excessively high (Weilu Huang: 18.82 m/s) or low (Tingting Liu: 14.95 m/s) preliminary swing speeds both disrupted the rotation radius, which is consistent with Hirose et al. (2017)’s view that “balanced preliminary swing speed is the basis for stable rotation”. This suggests that Chinese athletes should focus on controlling the preliminary swing speed to avoid the “double-edged sword” effect.

### Shoulder-hammer angle: Key to athlete-implement unity

The shoulder-hammer angle is a critical indicator of “athlete-hammer coordination” (Liang, 2008). Our study found that Chinese athletes (except Zheng Wang) had larger angles in later turns, which may lead to “back-hammer” movements and reduce rotation radius. In contrast, international athletes (e.g., DeAnna Price) maintained angles ≤90°, which is consistent with Dong et al. (2014)’s recommendation that “angles close to 90° facilitate lower limb force transfer”. Zheng Wang’s technique of keeping angles ≤90° at take-off further confirms Wang and Wu (2013)’s view that “optimizing shoulder-hammer angle shortens single-support phase and improves efficiency”.

### Summary of core findings and alignment with research objectives

This study identified four key technical gaps between Chinese and international elite female hammer throwers: (1) lower release velocity due to inefficient final exertion; (2) irregular rotational rhythms caused by imbalanced preliminary swing speeds; (3) longer but unstable trajectories reducing acceleration efficiency; (4) excessive shoulder-hammer angles impairing athlete-implement coordination.

These findings directly respond to the study’s purpose of “exploring technical differences to provide data support for Chinese athletes’ training optimization”. By comparing with international literature, we clarified that the technical optimization of Chinese athletes should focus on: improving explosive power in the final phase, balancing preliminary swing speed to stabilize rotation rhythm, refining trajectory consistency, and controlling shoulder-hammer angle to enhance force transfer. These strategies can strengthen the scientific basis of Chinese women’s hammer throw training and promote the narrowing of the gap with world-class athletes.

## Limitations

This study focused on elite hammer throwers and did not include junior athletes, making it difficult to clarify the developmental stages of technical evolution. The current findings are mainly based on experimental data and observational analyses from specific time periods, focusing on short-term performance and changes in certain technical aspects. However, the long-term development of hammer throw technique is a dynamic and evolving process influenced by innovations in training methods, advances in materials technology, rule changes, and other factors. The current results only show the present technical features and patterns, lacking a thorough and systematic discussion on how these might influence or limit the long-term development of hammer throw technique or whether they could cause fundamental changes in the technical system. Furthermore, the video recording methods used faced technical limitations and potential biases. During data collection, the video equipment was restricted by resolution, frame rate, and other factors. Psychological factors (e.g., Tingting Liu’s abnormal release angle, possibly linked to pre-competition anxiety) and environmental variables (e.g., wind speed, equipment differences) were not taken into account. Future research should develop integrated assessment models that incorporate these factors and it is recommended to conduct longitudinal technical tracking of athletes to analyse correlations between technical improvements and performance over multiple years.

## Conclusion

The international elite female hammer throwers exhibit superior release-related indices and more effective double-support mechanics compared with their Chinese counterparts. These results highlight the central role of double-support coordination, trunk–pelvis coupling, and phase-specific timing in optimizing release velocity and overall performance. Targeted technical interventions focusing on these elements may help close the performance gap and advance elite-level outcomes.

## Data Availability

The raw data supporting the conclusions of this article will be made available by the authors, without undue reservation.
